# The Protective Association of Trait and Ability Emotional Intelligence with Adolescent Tobacco Use

**DOI:** 10.3390/ijerph17186865

**Published:** 2020-09-20

**Authors:** Sara González-Yubero, Susana Lázaro-Visa, Raquel Palomera Martín

**Affiliations:** Education Department, University of Cantabria, 39005 Santander, Spain; susana.lazaro@unican.es (S.L.-V.); raquel.palomera@unican.es (R.P.M.)

**Keywords:** emotional intelligence, self-report, performance test, tobacco, adolescence

## Abstract

The present study is one of the first to analyze the predictive capacity of both trait and ability Emotional Intelligence (EI) based on the Mayer and Salovey model, in relation to tobacco use in a sample of Spanish adolescents. In this study, 799 students between the ages of 12 and 16 participated. A self-report on trait EI, an EI peak performance test, and questions about habits relating to tobacco use were administered. This cross-sectional study developed a quantitative and correlation-type methodology. The main results of the regression analyses, once the sex and age of the participants were controlled, revealed negative associations between the factors of clarity and emotional repair of the trait EI with respect to the variables of tobacco use, and a positive association was found for them and emotional attention. By comparison, with respect to ability EI, emotional perception and understanding were inversely related to adolescent tobacco use. These results underscore the importance of EI skills as protective factors against early initiation and subsequent tobacco abuse.

## 1. Introduction

As reported by international bodies, tobacco use is currently one of the greatest public health problems [1]. According to a recent study [2], the rate of smoking remains high among adolescents and, in recent years, the number of new smokers among the youth that are not yet of age (11–15 years) has increased. Smoking is the leading preventable cause of death and the most prominent modifiable risk factor for several diseases. Despite its harmful nature, it is a very appealing habit for young people, who are particularly vulnerable to nicotine addiction and the adverse effects of tobacco [3]. According to data from the latest Spanish Survey on Drug Use among Secondary Students [4], tobacco is the second most widely used drug among adolescents aged 14 to 18 after alcohol. Forty-one percent said they had smoked at some point, 27% in the last month, and of these, about one third (9.8%) were daily users. Likewise, the average age of onset is around 14 years, with comparable levels of use among men and women, and increasing progressively with age.

Adolescence is a complex developmental stage in which changes that promote the transition from childhood to adulthood occur in the physical, psychological, and social spheres [5]. This makes young people particularly vulnerable to certain risk behaviors such as drug use [6]. Prevalence studies show that tobacco use begins early, at the ages of 12 and 13 [7]. Thus, many smokers begin their addiction in adolescence [8], which makes understanding risk and protective factors at this stage of life vitally important. Longitudinal research has emphasized smoking among peers as one of the major risks of adolescent use [9]. Similarly, numerous studies have pointed out the relationship between the frequency and quantity of tobacco use and the harmful effect on the physical health of adolescents [10], depressive symptoms [11], suicidal ideation [12], and other addictive behaviors such as heavy alcohol consumption [13], cannabis [14], and gambling addiction [15].

The literature has identified several risk factors for substance use among young people, including family, school, community, and social and personal factors [16]. As for the latter, associations have been found between tobacco use, a lack of impulse control, and sensation-seeking [17], alexithymia [18], deficient emotional skills [19,20,21]; maladaptive coping mechanisms [22], neuroticism [23], low self-esteem [24], and low feelings of self-efficacy among others [25]. Based on this, it can be concluded that the risk of tobacco and other substance use at an early age presents clear emotional consequences.

Of the protective factors for substance use, special attention should be paid to emotional intelligence (EI), the promotion of which at young ages may serve as a novel preventive measure [26,27,28,29]. EI is regarded as a factor in promoting public health [30] and preventing psychosocial maladjustment in adolescence [31,32,33,34]. Research has shown that EI, understood as the ability to recognize, understand, and regulate one’s own and other people’s emotions, to discriminate between them and then use the information to guide thoughts and actions [35], is a predictor of alcohol, tobacco, and illegal substance use mostly in adults and university students [36,37]. However, it should be noted that very few studies have examined the association between trait and ability EI with respect to tobacco use in adolescents, which would provide new evidence to advance in the clinical and educational field. 

Since the concept of EI was initially formulated, growing interest in the field has led to numerous evaluation methods [38]. Currently, two EI constructs can be identified based on the method of measurement used, with the literature being compiled independently [39]. First, the trait EI refers to the self-perception of a series of emotional aptitudes evaluated via self-reports pertaining to the field of personality. Second, the ability EI references the cognitive capacity to correctly respond to various emotional tasks through peak performance tests. Although self-report measures of EI rely on the subject’s perception of their own emotional abilities, the model proposed by Mayer and Salovey [35] stresses the importance of using maximum performance measures to assess the real ability of the person, thus following the traditional methodology used to measure cognitive intelligences [40,41].

Among the studies that have evaluated the relationship of tobacco use and the trait EI, one study found that those college students who were heavier smokers presented low levels of the emotional repair component and began smoking at a younger age [42]. Results from other research with adolescents showed that those with a higher perceived ability to repair their negative emotions smoked less [7]. On the other hand, excessive attention to emotional states was associated with increased use [43]. The authors concluded that greater attentiveness to feelings together with an inability to understand and regulate emotions may lead to increased ruminative thinking and thus facilitate increased use. In another study [44], conducted with 16–65-year-olds, the authors concluded that non-regular smokers had greater clarity of emotion and a greater ability to repair negative emotional states than regular smokers. Although the literature has supported a link between higher levels of EI, superior physical and mental health, and reduced tobacco use in adults [45,46], the idea that access to addictive substances is more difficult for adolescents has received some support [47], and, therefore, few studies have focused on this age range.

On the other hand, with regard to studies that evaluated EI as an ability using maximum performance tests, [7] employed an emotional perception performance task and found that adolescents with a greater ability to perceive the emotions of others reported lower use of this substance. Likewise, [48] found negative associations between total EI and the ability to perceive and understand emotions with respect to the intensity of tobacco use and earlier initiation among adolescents [48]. In a second study by this group, higher EI was associated with greater perception of negative social consequences associated with tobacco use (e.g., “cigarette smoking is a way of losing non-smoking friends”), increased efficiency in rejecting the offer of cigarettes by peers, and a lesser intention to smoke in the coming year [49].

Given that the literature warns that tobacco use begins at an early age [7], and most studies have been conducted on adults and university students, additional empirical evidence on the role of EI in adolescent tobacco use may be especially useful in orienting new preventive actions. In this sense, given that the abilities included in EI can be learned and improved by avoiding health-risk behaviors [41], we consider that this study could guide the design of clinical and educational interventions aimed at preventing the appearance of problems involving psychosocial imbalance in adolescence such as drug use, in particular tobacco. Likewise, many studies have only used EI self-reporting measures and virtually none have applied maximum performance tests, which would permit a greater understanding of its role in this issue. According to the previous literature and given that ESTUDES [4], uses a limited sampling frame that includes only adolescents from 14 to 18 years of age, this research adds empirical data regarding the prevalence of consumption among the youngest (12–13 years). Given current concerns regarding the adverse effects of tobacco during vulnerable periods such as adolescence, this paper aims to analyze the relationship between the dimensions of trait EI and ability across various variables of use in students aged 12 to 16 years. Based on the results of previous studies, the following hypothesis has been described:

**Hypothesis 1** **(H1).** The dimensions of the trait and ability EI will be inversely and significantly correlated with the tobacco use dependent variables (“having tried tobacco before”; “number of days of tobacco use in the last year”; “number of cigarettes smoked per week”; “tobacco use when offered by friends”), with the exception of the factor of attention to one’s own emotions of the trait EI, which will be positively and significantly correlated with the same.

**Hypothesis 2** **(H2).** After controlling for sex and age, the dimensions of the trait and ability EI will be inversely and significantly associated with the tobacco-use dependent variables with the exception of the factor of attention to one’s own emotions of the trait EI, which will be positively and significantly associated with the same.

## 2. Materials and Methods

### 2.1. Participants

844 Spanish students of Obligatory Secondary Education (ESO) with the ages between 12 and 16 participated in this research. Stratified random sampling was applied according to the school ownership (charter 50%, public 50%), the rural (30%) or urban (60%) context and an equal representation of sex (50% male and 50% female) and age (preadolescents 40% and adolescents 60%) in accordance with the proportion present in the reference population. Of the educational centers initially selected, 66.6% participated. Exclusion criteria included being outside the age range of 12–16 years (*N* = 21), as well as not filling out the questionnaire in full by the end of the second session (*N* = 24). The final sample was 94.6% with respect to the initial sample (*N* = 799) and was composed of adolescents between the ages of 12 and 16 (12–13 years 38.2%; 14–16 years 61.8%; *M* = 14.49; *SD* = 1.17), with a balanced sex distribution (51.8% women and 48.2% men). Fifty-one percent attended charter schools and 48.6% attended public schools, of which 64% were in urban areas and 36% in rural areas.

### 2.2. Instruments

Dependent variables: Tobacco use questionnaire. An adaptation of 4 items from ESTUDES [4] was used: “previous tobacco use” (I have tried tobacco before/I have never tried tobacco), “tobacco use when offered by friends” (I accepted and smoked/I did not accept or smoke), “number of days of tobacco use in the last 12 months” (1–7, 8–14, 15–21, 22–28, 29–35, 36–42, 43–49, 50 or more) and “quantity of weekly units” (1–5, 6–10, 11–15, 16–20, 21–25, 26–30, 31–35, 36–40, 41 or more).

Control variables: Socio-demographic data questionnaire. Sex (female/male) and age (12–13/14–16 years old) were collected.

Independent variables: Trait Meta-Mood Scale [50]. Spanish validation by [51]. It consists of 24 items and provides an indicator of the levels of the emotional intelligence trait. It is composed of three factors: “emotional attention” (e.g., “I pay a lot of attention to my feelings”); “emotional clarity” (e.g., “I often misread my feelings”); and “emotional repair” (e.g., “although I feel sad occasionally, I usually have an optimistic outlook”). Its items are assessed using a 5-point Likert-like scale ranging from strongly disagree (1) to strongly agree (5). The Cronbach’s alpha for this sample was 0.87 for attention, 0.85 for clarity, and 0.82 for emotional repair. 

Test of emotional intelligence by the Fundación Botín for adolescents TIEBDA; original Spanish validation by [52]. It provides measures of performance levels in each of the four emotional abilities of Mayer and Salovey‘s theoretical model [35]. It consists of 144 items that present eight emotional scenarios featuring various adolescent characters. The instrument is classified in four dimensions: “Emotional perception” (48 items e.g., “to what extent do you think Rocío expresses each of the following feelings?”); “emotional facilitation” (24 items e.g., “to what extent will feeling this way help Rocío to review the list of school materials she needs to buy this year”); “emotional understanding” (32 items e.g., “what might Rocío be thinking that is causing her to feel this way?”); and “emotional regulation” (40 items e.g., “what can Rocío do in order to leave school happy?”). Its items are assessed using a 5-point Likert-like scale ranging from strongly disagree (1) to strongly agree (5). The Cronbach’s alpha for this sample was 0.86 for perception, 0.78 for facilitation, 0.80 for understanding, and 0.76 for emotional management.

### 2.3. Procedure

This research is governed by the principles set out in the Declaration of Helsinki [53]. Likewise, the development of the research plan for the present study was presented to the Academic Commission for Doctoral Studies of the University of Cantabria. An informed-consent document addressed to the centers and the students’ legal representatives was prepared to obtain their signed authorization prior to the students’ participation in the study. In addition, the student’s informed consent was collected in each of the educational centers. The questionnaires were administered in the classroom. Students filled all questionnaires at their own classes in the presence of a researcher. The time required to complete the instrument using a pen and paper was two non-consecutive 45 min sessions. Moreover, numerical codes were used in each of the questionnaires as a means of keeping the identities of the students confidential. Furthermore, once the test was completed, all the questionnaires were collected in sealed envelopes.

### 2.4. Data Analysis

The statistical package IBM SPAIN SPSS Statistics 24.0 version was used for data analysis processing. The study was conducted using a quantitative and correlational methodology. First, Cronbach’s alpha reliability indices were calculated for each of the EI factors evaluated. After that, descriptive analyses were carried out to observe the prevalence of tobacco consumption (Table 1). To test Hypothesis 1, point bias correlation analyses of the study variables were performed (Table 2). Subsequently, to test Hypothesis 2, binary logistic regression models were developed based on the factors of trait EI (attention, clarity and emotional repair) and ability EI (perception, facilitation, understanding, and emotional regulation) also taking into account the effect of age and sex when observing their association with the dependent variables: (1) “having tried tobacco before”; (2) “tobacco use when offered by friends’’; (3) “frequency of tobacco use per year”; (4) “number of cigarettes smoked per week”. The dependent variables 3 and 4 were dichotomized by the median. Finally, the procedure used was designed to introduce into the models the factors that presented significant bivariate correlations in the previous analyses. The backward step method was used, extracting the variables one by one until arriving at a model where all the predictors were significant (at least *p* <0.05); see Table 3. To synthesize the amount of data, only the final models that explain a higher percentage of variance were presented.

## 3. Results

### 3.1. Prevalence of Adolescent Tobacco Use

Table 1 presents the data in relation to the prevalence of tobacco use in the study sample. Of initial interest is that approximately a quarter of the adolescents had tried tobacco at some point in their lives (25.7%). Among smokers, approximately 6 out of 10 had smoked 40 or more days in the previous year. Also, about 6 in 10 reported smoking 20 or more cigarettes per week. Finally, around one fifth of the total sample claimed to smoke when offered by a friend (21.9%). In relation to the socio-demographic variables, 2 out of 10 women stated that they had smoked at least once in their lives, while the prevalence among men was slightly higher (28.1%). With respect to age group, approximately 2 out of 10 school children aged 12–13 years claimed to have used tobacco, with an appreciable increase in use in the group aged 14–16 years (30.4%).

### 3.2. Correlation Analysis between Trait and Ability EI and Tobacco Use

The results of the correlation analysis are shown below (Table 2). With respect to H1, there were significant negative correlations between trait and ability EI and tobacco use variables, except for attention to one’s own feelings, which showed a significant direct correlation. The effect size of the correlations was mainly low [54]. For the variable “have tried tobacco before” only, the effect size was medium, with the factors of clarity and emotional repair. On the other hand, correlations with understanding and repair factors were highlighted in relation to the frequency of “annual tobacco use”. For “number of cigarettes smoked per week” the highest correlations were obtained with understanding and repair. Finally, regarding the variable “tobacco use when offered by friends”, the highest correlations were found with the factors of repair and clarity.

### 3.3. Binary Logistic Regression of Tobacco Use According to Trait and Ability EI, Sex, and Age

In relation to H2, binary logistic regression analyses were carried out to study the contribution of EI with respect to tobacco consumption once the sex and age of the adolescents were controlled. To synthesize the abundant amount of data, only the final models that proved to be statistically significant and that accounted for a larger percentage of the variance have been presented in this section. To begin with (Table 3), the model created for the dependent variable “having tried tobacco before” allowed for a correct estimate in 74.1% of cases. The independent variables of attention, clarity, repair, and perception formed part of the equation. The odds ratio obtained for each variable indicates that: (a) the probability of being a tobacco user is 1.03 times higher among adolescents with a high level of emotional attention than those with a lower level; (b) the probability of being a tobacco user is 0.92, 0.95, and 0.96 times higher, respectively, among adolescents with less ability to understand, repair, and perceive emotions than those with higher skills. Nagelkerke R^2^ statistic estimated an adjustment value of 0.286. Secondly, when taking “frequency of tobacco use per year” as a criterion variable, a correct estimate of the model was obtained in 76.2% of the cases, with the independent variables of age, repair, and emotional understanding forming part of the equation. The odds ratio obtained for each variable indicate that: the probability of having smoked for 40 days or more during the last year is 4.48, 0.93, and 0.96 times higher, respectively, among adolescents between 14–16 years with less ability to repair and understand emotions than those with higher skills. Nagelkerke R^2^ statistic estimated an adjustment value of 0.281. For the dependent variable “number of cigarettes smoked per week”, the model allowed for a correct estimate in 74.4% of cases, with age, repair, and understanding forming part of the equation. The odds ratio obtained for each variable indicate that the probability of having smoked 20 or more cigarettes a week is 6.94, 0.94, and 0.96 times higher, respectively, among adolescents between 14–16 years with less ability to repair and understand emotions than those with higher skills. Nagelkerke R^2^ statistic estimated an adjustment value of 0.335. Finally, for the dependent variable “tobacco use when offered by friends”, the model allowed for a correct estimate in 79.4% of cases, with clarity, repair, and understanding forming part of the equation. The odds ratio obtained for each variable indicate that the probability of smoking when a friend offers is 0.96, 0.94, and 0.97 times higher, respectively, among adolescents with less ability to understand, repair, and perceive emotions than those with higher skills. Nagelkerke R^2^ statistic estimated an adjustment value of 0.182. 

As indicated (Table 3), the socio-demographic variable of age proved to be significant for the “frequency of tobacco use per year” model, as well as for the “number of cigarettes smoked per week” model. The results are then stratified according to the two age groups for both usage variables (Table 4 and Table 5).

### 3.4. Binary Logistic Regression of Tobacco Use as a Function of Trait and Ability EI for Each Age Group (12–13 Years and 14–16 Years)

First, the model created for “frequency of tobacco use per year” with the group of 12–13-year-olds allowed for a correct estimate in 77.2% of the cases. The independent variables of repair and understanding formed part of the equation. The odds ratio obtained for each variable indicate that: the probability of having smoked for 40 days or more during the last year is 0.91 and 0.93 times higher, respectively, among adolescents with less ability to repair and understand emotions than those with higher skills. Nagelkerke R^2^ statistic estimated an adjustment value of 0.324. Likewise, the model for the age group of 14–16 years allowed for a correct estimate in 72.6% of the cases. The independent variables of repair and emotional understanding formed part of the equation. The odds ratio obtained for each variable indicate that the probability of having smoked 20 or more cigarettes a week is 0.91 times higher among adolescents with less ability to understand emotions than those with a higher skill. Nagelkerke R^2^ statistic estimated an adjustment value of 0.115. Secondly, the model for the dependent variable “number of cigarettes smoked per week” with respect to the age group of 12–13 years allowed for a correct estimate in 73.2% of the cases. The independent variable of emotional understanding formed part of the equation. The odds ratio obtained for each variable indicate that the probability of having smoked for 40 days or more during the last year is 0.91 and 0.93 times higher, respectively, among adolescents with less ability to repair and understand emotions than those with higher skills. Nagelkerke R^2^ statistic estimated an adjustment value of 0.269. Finally, for the age group of 14–16 years, the model allowed for a correct estimate in 72.2% of the cases. The emotional understanding variable formed part of the equation. The odds ratio obtained for each variable indicate that: the probability of having smoked 20 or more cigarettes a week is 0.97 times higher among adolescents with less ability to understand emotions than those with a higher skill. Nagelkerke R^2^ statistic estimated an adjustment value of 0.149.

The present study offers revealing data on the relationship between EI and tobacco use after controlling for sex and age in a sample of Spanish adolescents. First of all, in accordance with previous literature on the field of EI and drug consumption in adolescence [19,27,28], the effect size of the correlations was mainly low, although medium effect size was found between the variable “have tried tobacco before” and the factors of clarity and emotional repair. Secondly, the findings of this research support that both the components of clarity and emotional repair trait EI and the factors of perception and understanding of ability EI were inversely associated with tobacco use, as opposed to emotional attention, which was directly associated. In particular, the probability of being a tobacco user was higher among adolescents with a high level of emotional attention than those with a lower level. On the other hand, the risk of being a tobacco user, having smoked more frequently during the last year, as well as smoking more cigarettes per week and smoking when friends offer was higher among adolescents with less ability to understand, repair, and perceive emotions properly than those with higher skills. The role of repair and emotional understanding on the frequency and quantity of tobacco use in each age group after the segmented analyses was especially noteworthy. Likewise, even though the factors of facilitation and emotional regulation of ability EI were not explanatory for tobacco use, they did in fact correlate with some of the variables for the use of this substance. Finally, based on descriptive analyses, it can be sustained that the prevalence of tobacco use in students coincides in general terms with the latest Survey on Drug Use in Secondary School Students [4], demonstrating the need for preventive educational interventions from an early age.

First, with respect to the results on the trait EI factors, it can be concluded that emotional clarity and repair were negatively associated with occasional tobacco use and with a greater propensity to smoke when offered by friends. Likewise, a lower perceived ability to repair negative emotional states was associated with a higher frequency and quantity of tobacco use among adolescents. It is worth mentioning that once the results were stratified by age, emotional repair was negatively associated with the frequency of use per year in both age groups. These findings are consistent with those from other research in which the intensity of tobacco use increased in young people with less perceived ability to understand and regulate their emotional states [7,42,43]. Peer pressure is one of the factors most closely associated with the onset of drug use and abuse [13]. As in other past studies [48], these results support the idea that adolescents who are more capable of understanding and regulating their own emotions and those of others are more competent when it comes to managing disagreements with the group, which in turn reduces their risk of consumption due to pressure from the same.

Based on the data obtained, adolescents who scored higher in “attention to their own emotions” were more likely to have used tobacco at some point in their lives. Therefore, several past studies have pointed out the implication between high levels of emotional attention, tobacco use and other addictive substance use among adults and adolescents [7,19,27,43,55]. A high level of emotional attention has been consistently associated in a positive manner with anxiety, depression, and maladjusted coping strategies in past literature [56,57]. Therefore, the tendency to focus one’s attention on one’s own emotional states allows the process of one’s emotions to be followed, but this is not always adaptive. High levels of attention to one’s emotional states could lead to an increase in ruminative thinking and an unpleasant mood [43]. Therefore, it is possible that youth with heightened attention to their feelings who are unable to understand and regulate their emotions choose to use tobacco as a means of mitigating their adverse emotional states.

As for ability EI factors, the ability to properly perceive and understand emotions was inversely associated with tobacco use variables. Thus, a lower emotional perception was related to a higher probability of having tried tobacco and of having done so when presented with the offer by friends. These results are consistent with the study by [7], which found that a weaker ability to perceive emotions predicted increased tobacco use in adolescents. On the other hand, emotional understanding was negatively associated with frequency of use per year and number of cigarettes per week. The important role of this ability with respect to frequency and quantity of use for both age groups in this study should be highlighted. Our findings are consistent with other works that corroborate the protective function of emotional perception and understanding abilities on tobacco use at young ages [7,48].

## 4. Conclusions

Despite the importance of facilitation and regulation abilities in promoting proper cognitive processing and regulating emotions, these factors were not explanatory for tobacco use variables, although they had a significant inverse correlation with certain variables. The data obtained in this research support the idea that lower levels of EI are related to a higher probability and frequency of tobacco use, as well as a higher quantity and use when offered by friends. Therefore, it could be concluded that those adolescents with a greater capacity to perceive emotions and attend to theirs in a moderate way, and who are capable of understanding and repairing their own negative emotional states, are less likely to initiate and increase tobacco use.

The results of this research highlight the importance of developing these abilities through drug prevention programs as a protective factor against early onset and eventual substance abuse. We therefore consider that preventive interventions could increase effectiveness if they take into account the building of abilities needed to perceive, understand, and regulate emotions, as well as the interpersonal abilities needed to withstand pressure from friends when it comes to tobacco use. Finally, the results obtained in this study provide a foundation for the development of future lines of research that can further advance the understanding of the variables involved in adolescent tobacco use. The results obtained are aligned with scientific advances that underline the need to consider the training of social and emotional skills in various contexts of the preventive field, such as the family, educational, work, and community [1,58]. In addition, although the risk factors associated with consumption are numerous and many of them cannot be modified, the emotional skills included in this study can be learned and improved acting as protective factors against drug consumption in adolescents [59]. For all these reasons, we consider that the findings of this research offer empirical support to prevention programs focused on the promotion of personal resources to improve these skills.

We believe that further studies should validate the results obtained here. Causal inferences from this research should be treated with caution due to its cross-sectional design. It would therefore be of interest for longitudinal studies with heterogeneous samples to further corroborate these results. Similarly, it would be interesting to study the directionality between EI and tobacco use, as well as the effect that other variables such as attitudes towards smoking and coping strategies have at these ages. Despite these limitations, this research provides additional information on the relationship between EI and tobacco use in the earliest stage of adolescence (12–16 years), of which very little has been studied. Also, given that research combining the assessment of the constructs of trait EI and ability EI is difficult to find, this study provides a more complete understanding of their role in tobacco use.

## Figures and Tables

**Table 1:** Prevalence of Tobacco Use.

Tobacco	Response Categories	Percentage	*N*
Have tried tobacco	no, I have never tried tobacco	74.3	593
	yes, I have tried tobacco	25.7	206
Smoking frequency per year	<40 days	35.8	74
	≥40 days	64.1	132
Number of cigarettes per week	<20 cigarettes	38.9	79
	≥20 cigarettes	61.1	124
Use when offered by a friend	did not smoke	78.1	623
	smoked	21.9	176
Use by sex	female no	76.8	308
	female yes	23.3	93
	male no	71.9	286
	male yes	28.1	113
Use by age	12–13 years old no	82.0	251
	12–13 years old yes	18.8	55
	14–16 years old no	69.6	353
	14–16 years old yes	30.4	151

**Table 2:** Biserial-Point Correlation between Trait and Ability EI Variables and Tobacco Use.

Emotional Intelligence	Have Tried Tobacco Before	Previous Year	Cigarettes Weekly	Usage When Offered by Friends
1. TMMS Attention	0.21 **	0.16 *	0.16 *	0.16 *
2. TMMS Clarity	−0.34 **	−0.20 **	−0.18 *	−0.25 **
3. TMMS Repair	−0.33 **	−0.26 **	−0.23 **	−0.27 **
4. TIEBDA Perception	−0.28 **	−0.24 **	−0.20 **	−0.23 **
5. TIEBDA Facilitation	−0.16 *	−0.05	−0.06	−0.18 **
6. TIEBDA Understanding	−0.24 **	−0.27 **	−0.29 **	−0.19 **
7. TIEBDA Managing	−0.15 *	−0.11	−0.11	−0.12 *

Note: TMMS = Trait Meta-Mood Scale; TIEBDA = Test of emotional intelligence by the Fundación Botín for adolescents. * = *p < 0*.05. ** = *p < 0*.01.

**Table 3:** Binary Logistic Regression of Tobacco Use as a Function of Trait and Ability EI.

Tobacco and Emotional Intelligence Variables	B	S.E.	Wald	OR	IC 95% of the OR
Have tried tobacco before					
Attention (TMMS)	0.03	0.01	5.72 **	1.03	1.00/1.06
Clarity (TMMS)	−0.08	0.02	22.32 **	0.92	0.89/0.95
Repair (TMMS)	−0.05	0.02	8.94 **	0.95	0.92/0.98
Perception (TIEBDA)	−0.04	0.01	24.57 **	0.96	0.95/0.98
constant	4.255	1.08	15..51 **	--	--
Frequency of use per year					
Age	1.50	0.36	17.56 **	4.48	2.22/9.03
Repair (TMMS)	−0.07	0.02	11.03 **	0.93	0.89/0.97
Understanding (TIEBDA)	−0.04	0.01	12.47 **	0.96	0.94/0.98
constant	3.400	1.47	5.33 *	--	--
Number of cigarettes per week					
Age	1.94	0.37	26.63 **	6.94	3.23/14.49
Repair (TMMS)	−0.06	0.02	8.44 **	0.94	0.89/0.98
Understanding (TIEBDA)	−0.04	0.01	14.63 **	0.96	0.93/0.99
constant	2.640	1.48	3.17 *	--	--
Use by offer of a friend					
Clarity (TMMS)	−0.04	0.02	7.19 **	0.96	0.93/0.99
Repair (TMMS)	−0.06	0.02	11.95 *	0.94	0.91/0.97
Perception (TIEBDA)	−0.03	0.01	21.15 **	0.97	0.95/0.98
constant	3.938	0.97	33.32 **	--	--

Note: TMMS = Trait Meta-Mood Scale; TIEBDA = Test of emotional intelligence by the Fundación Botín for adolescents. *B* = coefficient; S.E. = standard error; OR = odds ratio; C.I. = confidence interval; * = *p < 0*.05; ** = *p < 0*.01.

**Table 4:** Binary Logistic Regression of Tobacco Use as a Function of Trait and Ability EI for the 12–13-Year-Old Group.

Tobacco and Emotional Intelligence Variables	B	S.E.	Wald	OR	IC 95% of the OR
Smoking frequency per year					
Repair (TMMS)	−0.09	0.04	4.76 *	0.91	0.83/0.99
Understanding (TIEBDA)	−0.07	0.03	6.08 **	0.93	0.88/0.98
constant	8.06	2.99	8.04 **	--	--
					
Number of cigarettes per week					
Understanding (TIEBDA)	−0.09	0.03	7.60 **	0.91	0.85/0.97
constant	8.32	3.27	6.49 **	--	--

Note: TMMS = Trait Meta-Mood Scale; TIEBDA = Test of emotional intelligence by the Fundación Botín for adolescents. *B* = coefficient; S.E. = standard error; OR = odds ratio; C.I. = confidence interval; * = *p < 0*.05; ** = *p < 0*.01.

**Table 5:** Binary Logistic Regression of Tobacco Use as a Function of Trait and Ability EI for the 14–16-Year-Old Age Group.

Tobacco and Emotional Intelligence Variables	B	S.E.	Wald	OR	IC 95% of the OR
Smoking frequency per year					
Repair (TMMS)	0.06	0.02	5.46 *	0.91	0.90/0.99
Understanding (TIEBDA)	−0.03	0.01	6.53 **	0.93	0.94/0.99
constant	5.389	1.42	14.45 **	--	--
					
Number of cigarettes per week					
Understanding (TIEBDA)	−0.03	0.01	6.04 **	0.97	0.95/0.99
constant	5.749	1.54	13.91 **	--	--

Note: TMMS = Trait Meta-Mood Scale; TIEBDA = Test of Emotional Intelligence by the Fundación Botín for Adolescents. *B* = coefficient; S.E. = standard error; OR = odds ratio; C.I. = confidence interval; * = 4. Discussion.
